# Association Between Three Systemic Inflammatory Biomarkers and Diabetic Foot Ulcer: A Cross‐Sectional Study and a Clinical Retrospective Study

**DOI:** 10.1155/mi/7709529

**Published:** 2026-02-19

**Authors:** Hua Chen, Yu Zhou, Jiezhi Dai

**Affiliations:** ^1^ Department of Orthopedic Surgery, Shanghai Sixth People’s Hospital, JiaoTong University, Shanghai, China, sjtu.edu.cn; ^2^ Department of Orthopedic Surgery, Civil Aviation Hospital of Shanghai, Shanghai, China

**Keywords:** aggregate index of systemic inflammation, DFU, NHANES, retrospective study, system immune inflammation index, systemic inflammatory response index

## Abstract

**Background:**

This study aimed to investigate the association between systemic inflammatory biomarkers—specifically the systemic inflammatory response index (SIRI), systemic immune inflammation index (SII), and aggregate index of systemic inflammation (AISI)—and the prevalence of diabetic foot ulcer (DFU).

**Method:**

We conducted a cross‐sectional analysis using data from three cycles of the National Health and Nutrition Examination Survey (NHANES), supplemented by a single‐center retrospective clinical study. Initially, 31,126 participants were screened from NHANES. Binary logistic regression models (both unadjusted and adjusted for covariates) were employed to evaluate the associations between ln SIRI, ln SII, and ln AISI and DFU prevalence. Restricted cubic spline (RCS) analysis was applied to assess nonlinear relationships, and subgroup analyses were performed to examine the stability of associations across strata defined by age, gender, race, body mass index (BMI), smoking status, and hypertension. Additionally, a clinical validation study was conducted from January to December 2023, comprising 34 DFU patients and 68 diabetic controls. We performed multivariable binary logistic regression analyses to assess the independent associations between systemic inflammatory indices and the presence of DFU, adjusting for age, sex, diabetes duration, BMI, and albumin levels. Receiver operating characteristic (ROC) curve analysis was used to preliminarily assess the discriminatory ability of SII, SIRI, and AISI for DFU status.

**Results:**

The cross‐sectional analysis included 135 participants with DFU and 1560 without DFU. Logistic regression revealed consistent positive associations between ln SIRI, ln SII, and ln AISI and DFU prevalence in both unadjusted and adjusted models. RCS analysis indicated linear dose–response relationships for all three biomarkers. Subgroup analyses confirmed that these associations remained stable across demographic and clinical subgroups. In the retrospective clinical study, ln SIRI, ln SII, and ln AISI were significantly associated with DFU prevalence, with odds ratios (ORs) as follows: ln SIRI: OR = 2.51 (95% CI: 1.39–4.54), ln SII: OR = 5.44 (95% CI: 2.48–11.91), and ln AISI: OR = 2.75 (95% CI: 1.59–4.74). After adjusting for key confounders, the associations between these biomarkers and DFU remained consistent in both direction and statistical significance. ROC analysis showed that SII was more reliable than the other two for predicting DFU.

**Conclusion:**

By integrating a cross‐sectional NHANES–based analysis with a clinical retrospective study, this research demonstrates that elevated levels of SIRI, SII, and AISI are significantly associated with an increased prevalence of DFU. These systemic inflammatory biomarkers may serve as valuable tools for risk assessment in patients with DFU.

## 1. Introduction

Diabetic foot ulcer (DFU) represents one of the most severe and life‐threatening complications of diabetes [1]. It is described as ulcerated feet in diabetic patients with lower extremity neuropathy and/or peripheral artery disease. Chronic ulcers are the main cause of hospitalization and amputation in patients with diabetes, seriously affecting their quality of life. Consequently, early diagnosis and intervention for DFU are of critical clinical importance.

Emerging evidence highlights the pivotal role of chronic inflammation in DFU pathogenesis, characterized by a sustained pro‐inflammatory microenvironment dominated by cytokines including TNF‐α, IL‐1β, and IL‐6, which collectively impair angiogenesis and delay wound healing [2]. Traditional inflammatory biomarkers, such as white blood cell (WBC) count, C‐reactive protein (CRP), procalcitonin, and erythrocyte sedimentation rate (ESR), are significantly elevated in patients with DFU. However, their diagnostic utility remains limited by insufficient specificity and susceptibility to confounding factors.

Recently, novel systemic inflammatory biomarkers, including the systemic inflammatory response index (SIRI), systemic immune inflammation index (SII), and aggregate index of systemic inflammation (AISI) have emerged as new indicators based on neutrophil cells, lymphocytes, monocytes, and platelets [3]. These biomarkers have been widely used to evaluate the association between various chronic inflammatory status and human diseases [4]. Huang et al. [5] found significant positive correlations between SIRI and AISI with the incidence of heart failure. Song et al. [6] demonstrated that the levels of SII, SIRI, and AISI were higher in peripheral arterial disease in type 2 diabetic patients, and were independently linked with its clinical severity. Despite their utility as indicators of inflammatory status, these systemic inflammatory biomarkers have not been extensively studied in patients with DFU.

The National Health and Nutrition Examination Survey (NHANES), conducted by the Centers for Disease Control and Prevention (CDC) and the National Center for Health Statistics (NCHS), provides comprehensive national health data representative of the U.S. population [7]. To address the existing knowledge gap, this study employed a dual‐method approach: a cross‐sectional analysis utilizing NHANES data complemented by a clinical retrospective study, to comprehensively evaluate the association between SIRI, SII, and AISI and DFU prevalence.

## 2. Methods

### 2.1. Study Population and Study Design

**Figure 1:**
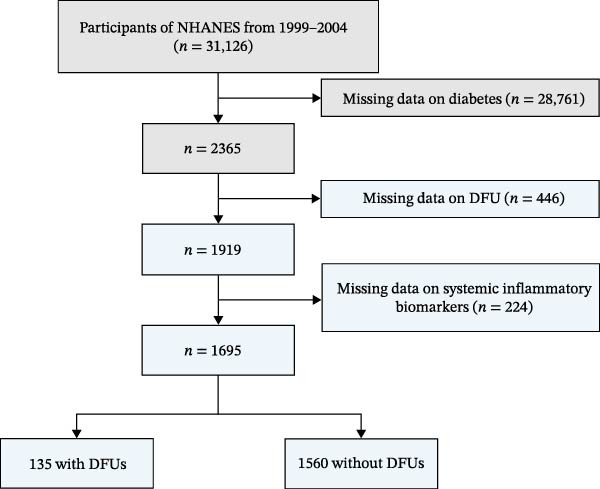
Flowchart of the participant selection.

This cross‐sectional analysis utilized data from three consecutive NHANES cycles (1999–2000, 2001−2002, and 2003–2004). From the initial pool of 31,126 participants, we applied the following exclusion criteria: missing data on diabetes status (*n* = 28,761), missing DFU information (*n* = 446), and missing values for systemic inflammatory biomarkers (*n* = 224). The final analytical cohort comprised 1695 individuals. Figure 1 illustrates the participant selection process. All NHANES study protocols received approval from the NCHS Research Ethics Review Board, and written informed consent was obtained from all participants.

### 2.2. Variable Determination

Demographic and clinical covariates included age, gender, race/ethnicity (categorized as Mexican American, other Hispanic, Non‐Hispanic White, Non‐Hispanic Black, and other race), education level (less than high school, high school, or more than high school), marital status, hypertension status, smoking history, body mass index (BMI), HbA1c, CRP, WBC count, and complete blood count (CBC) parameters (lymphocyte, monocyte, neutrophil, and platelet counts), as well as lipid profiles (total cholesterol and HDL‐cholesterol).

Diabetes mellitus was defined according to established criteria: (1) fasting blood glucose ≥126 mg/dL, (2) random blood glucose ≥200 mg/dL, (3) glycated hemoglobin (HbA1c) ≥6.5%, or (4) self‐reported physician diagnosis or current use of glucose‐lowering medications (insulin or oral agents). DFU was defined as the presence of foot ulceration persisting for more than 4 weeks, ascertained through structured diabetes questionnaires. Smoking status was categorized based on self‐reported history of having smoked at least 100 cigarettes in lifetime or currently smoking. Hypertension was defined as (1) average systolic blood pressure ≥140 mmHg, (2) average diastolic blood pressure ≥90 mmHg, or (3) self‐reported physician diagnosis or current antihypertensive medication use.

The systemic inflammatory biomarkers were calculated as follows:

SIRI=Neutrophil count×monocyte count/lymphocyte count.

SII=Platelet count×neutrophil count/lymphocyte count.

AISI=Neutrophil count×platelet count×monocyte count/lymphocyte count.

### 2.3. Statistical Analysis

All analyses were performed using SPSS version 18.0, R version 4.3.3, and Zstats 1.0 (www.zstats.net). Continuous variables were summarized as mean ± standard deviation, while categorical variables were expressed as frequencies and percentages. Group comparisons employed Student’s *t*‐test or Mann–Whitney test for continuous variables and chi‐square test for categorical variables, as appropriate.

Given the skewed distributions of SIRI, SII, and AISI, natural logarithmic transformations (ln SIRI, ln SII, and ln AISI) were applied for subsequent analyses. Multivariable binary logistic regression models were constructed to assess associations between systemic inflammatory biomarkers and DFU prevalence: Model 1: unadjusted; Model 2: adjusted for age, gender, and race/ethnicity; Model 3: adjusted for age, gender, race/ethnicity, BMI, smoking status, and hypertension.

Restricted cubic spline (RCS) regression was employed to evaluate potential nonlinear relationships between systemic inflammatory biomarkers and DFU prevalence, with adjustment for Model 3 covariates. Additionally, subgroup analyses were conducted to examine effect modification by age (<65 vs. ≥65 years), gender, race/ethnicity, BMI categories (<24, 24–28, ≥28 kg/m^2^), smoking status, and hypertension. The proportion of missing values was less than 5% across the dataset. To handle missing data, categorical variables were imputed using the most frequent category.

### 2.4. Clinical Validation Study

To clinically validate our findings, we conducted a single‐center retrospective study involving patients with DFU and matched type 2 diabetes controls without DFU, from January to December 2023. Type 2 diabetes was diagnosed according to the American Diabetes Association 2010 criteria [8]. DFU was defined in accordance with WHO as “ulceration of the foot (distally from the ankle and including the ankle) associated with neuropathy and varying degrees of ischemia and infection” [9]. We excluded patients with chronic wounds attributable to nondiabetic etiologies, including pyoderma gangrenosum, pressure ulcers, or vasculitis, to ensure a homogeneous study population specifically addressing diabetes‐related foot complications. We used a 1:2 individually matched case–control design. Controls were selected from the same patient database and were required to have type 2 diabetes without any history of DFU. To control for major confounders, each control was matched to a DFU case based on age (±5 years), and duration of known type 2 diabetes (±5 years). The matching was performed by reviewing electronic health records, selecting the two best‐matched controls for each case.

Study participants were categorized into DFU and non‐DFU groups. Baseline characteristics and laboratory parameters were compared between groups. Continuous variables were expressed as mean ± standard deviation and analyzed using Student’s *t*‐test, while categorical variables were presented as percentages and compared using chi‐square tests.

Due to nonnormal distributions, the systemic inflammatory biomarkers (SIRI, SII, and AISI) underwent natural logarithmic transformation (ln SIRI, ln SII, and ln AISI) for subsequent analyses. Multivariable binary logistic regression models was employed to evaluate the independent associations between these inflammatory indices and DFU prevalence. Model 1: unadjusted; Model 2: adjusted for age and gender; Model 3: adjusted for age, gender, diabetes duration, BMI, and albumin levels. Receiver operating characteristic (ROC) curve analysis was performed to preliminarily assess the discriminatory ability of SII, SIRI, and AISI for DFU status.

## 3. Results

### 3.1. Cross‐Sectional Study Findings

A total of 1695 participants were included in this cross‐sectional study according to the inclusion and exclusion criteria. In this study, 135 (7.99%) participants were diagnosed with DFU, and 1560 (92.01%) participants had diabetes without DFU. The average age was 64.48 years, and 876 (51.83%) individuals were males. In brief, there were significant differences between patients with DFU and those without DFU, including neutrophil, SIRI levels, SII levels, and AISI levels. Clinical characteristics were presented in Table 1.

**Table 1:** Baseline characteristics of the study population.

Characteristics	Total (*n* = 1695)	DFU (*n* = 135)	Non‐DFU (*n* = 1560)	*p*
Age (years)	—	65.10 ± 12.05	64.43 ± 11.86	0.530
Gender (%)				0.098
Male	876	79 (58.5%)	797 (51.1%)	—
Female	819	56 (41.5%)	763 (48.9%)	—
Race				0.631
Mexican American	478	42 (31.1%)	436 (27.9%)	—
Other Hispanic	72	6 (4.4%)	66 (4.2%)	—
Non‐Hispanic White	704	58 (43.0%)	646 (41.4%)	—
Non‐Hispanic Black	383	27 (20.0%)	356 (22.8%)	—
Other race	58	2 (1.5%)	56 (3.6%)	—
Education level				0.493
Less than high school	822	64 (47.4%)	758 (48.6%)	—
High school	351	24 (17.8%)	327 (21.0%)	—
More than high school	522	47 (34.8%)	475 (30.4%)	—
Marital status				0.095
Married/living with partners	1050	72 (53.3%)	978 (62.7%)	—
Widowed/divorced/separated	550	53 (39.3%)	497 (31.9%)	—
Never married	95	10 (7.4%)	85 (5.4%)	—
Smoking				0.813
Yes	900	73 (54.1%)	827 (53.0%)	—
No	795	62 (45.9%)	733 (47.0%)	—
Hypertension				0.444
Yes	1233	102 (75.6%)	1131 (72.5%)	—
No	462	33 (24.4%)	429 (27.5%)	—
BMI (kg/m^2^)	—	31.59 ± 7.82	30.64 ± 6.51	0.112
CRP (mg/dL)	—	0.84 ± 1.21	0.70 ± 1.51	0.306
WBC (10^9^)	—	7.78 ± 2.68	7.47 ± 2.16	0.085
Lymphocyte (10^9^)	—	2.04 ± 0.90	2.18 ± 0.94	0.100
Monocyte (10^9^)	—	0.61 ± 0.22	0.58 ± 0.19	0.100
Neutrophil (10^9^)	—	4.87 ± 2.10	4.46 ± 1.64	0.006
Platelet (10^9^)	—	259.90 ± 79.41	257.26 ± 73.87	0.691
Total cholesterol (mg/dL)	—	196.34 ± 45.28	203.51 ± 42.51	0.066
HDL‐cholesterol (mg/dL)	—	47.57 ± 16.64	48.18 ± 14.34	0.647
HbA1c (%)	—	7.29 ± 2.37	7.09 ± 1.85	0.283
SIRI	—	1.74 ± 1.48	1.36 ± 0.95	<0.001
SII	—	712.77 ± 466.54	605.48 ± 495.80	0.015
AISI	—	464.88 ± 459.68	354.42 ± 286.18	<0.001
ln SIRI	—	0.32 ± 0.65	0.13 ± 0.58	<0.001
ln SII	—	6.39 ± 0.59	6.24 ± 0.57	0.002
ln AISI	—	5.84 ± 0.75	5.64 ± 0.67	0.002

Abbreviations: AISI, aggregate index of systemic inflammation; SII, systemic immune‐inflammatory index; SIRI, systemic inflammatory response index.

Multivariable logistic regression analyses demonstrated consistent positive associations between logarithmically transformed inflammatory biomarkers (ln SIRI, ln SII, and ln AISI) and DFU prevalence across all models (Table 2). In the fully adjusted model (Model 3), which accounted for age, gender, race/ethnicity, BMI, smoking status, and hypertension, the odds ratios (ORs) were as follows: ln SIRI: OR = 2.03 (95% CI: 1.48–2.80), ln SII: OR = 1.78 (95% CI: 1.29–2.44), and ln AISI: OR = 1.70 (95% CI: 1.30–2.23).

**Table 2:** Association of ln SIRI, ln SII, and ln AISI with the incidence of DFU.

Variables	Model 1		Model 2		Model 3	
	OR (95% CI)	*p*	OR (95% CI)	*p*	OR (95% CI)	*p*
ln SIRI	1.70 (1.27, 2.29)	<0.001	2.02 (1.47, 2.77)	<0.001	2.03 (1.48, 2.80)	<0.001
ln SIRI tertiles						
Tertiles 1	1.00 (reference)	—	1.00 (reference)	—	1.00 (reference)	—
Tertiles 2	1.65 (1.03, 2.63)	0.038	1.60 (0.97, 2.63)	0.064	1.62 (0.98, 2.67)	0.060
Tertiles 3	1.96 (1.24, 3.11)	0.004	2.07 (1.25, 3.40)	0.004	2.07 (1.25, 3.42)	0.004
*p*‐trend	—	0.004	—	0.004	—	0.005
ln SII	1.62 (1.19, 2.20)	0.002	1.78 (1.29, 2.44)	<0.001	1.78 (1.29, 2.44)	<0.001
ln SII tertiles						
Tertiles 1	1.00 (reference)	—	1.00 (reference)	—	1.00 (reference)	—
Tertiles 2	1.09 (0.68, 1.72)	0.728	1.27 (0.78, 2.07)	0.332	1.27 (0.78, 2.06)	0.340
Tertiles 3	1.60 (1.04, 2.47)	0.032	1.82 (1.15, 2.88)	0.011	1.79 (1.13, 2.85)	0.013
*p*‐trend	—	0.027	—	0.010	—	0.012
ln AISI	1.52 (1.17, 1.97)	0.002	1.70 (1.30, 2.22)	<0.001	1.70 (1.30, 2.23)	<0.001
ln AISI tertiles						
Tertiles 1	1.00 (reference)	—	1.00 (reference)	—	1.00 (reference)	—
Tertiles 2	1.16 (0.72, 1.86)	0.546	1.27 (0.77, 2.09)	0.347	1.28 (0.78, 2.12)	0.330
Tertiles 3	1.89 (1.22, 2.93)	0.004	2.04 (1.28, 3.27)	0.003	2.03 (1.27, 3.26)	0.003
*p*‐trend	—	0.003	—	0.002	—	0.003

*Note*: Model 1, unadjusted model; Model 2, adjusted for sex, age, and race; Model 3, adjusted for sex, age, race, smoking, hypertension, and BMI.

Abbreviations: AISI, aggregate index of systemic inflammation; SII, systemic immune‐inflammatory index; SIRI, systemic inflammatory response index.

Tertile analysis further substantiated these findings. Participants in the highest tertile of ln SIRI, ln SII, and ln AISI exhibited significantly increased DFU risk compared to those in the lowest tertile. In Model 3, the corresponding ORs were: ln SIRI: OR = 2.07 (95% CI: 1.25–3.42), ln SII: OR = 1.79 (95% CI: 1.13–2.85), and ln AISI: OR = 2.03 (95% CI: 1.27–3.26). All trend tests yielded statistically significant results (*p* < 0.05).

**Figure 2:**
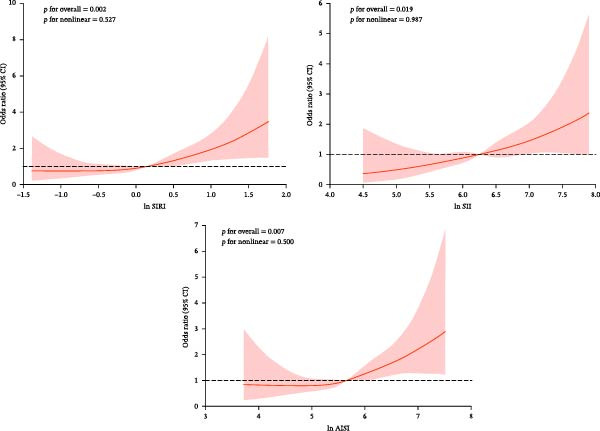
RCS analysis of ln SIRI, ln SII, and ln AISI with the prevalence of DFU.

Adjusting for confounding variables, RCS analyses showed ln SIRI, ln SII, and ln AISI were linearly positive with the prevalence of DFU (*p* for nonlinearity > 0.05; Figure 2).

RCS analyses revealed linear dose–response relationships between all three systemic inflammatory biomarkers and DFU prevalence, with nonlinearity tests indicating no significant deviation from linearity (*p* for nonlinearity > 0.05; Figure 2).

**Figure 3:**
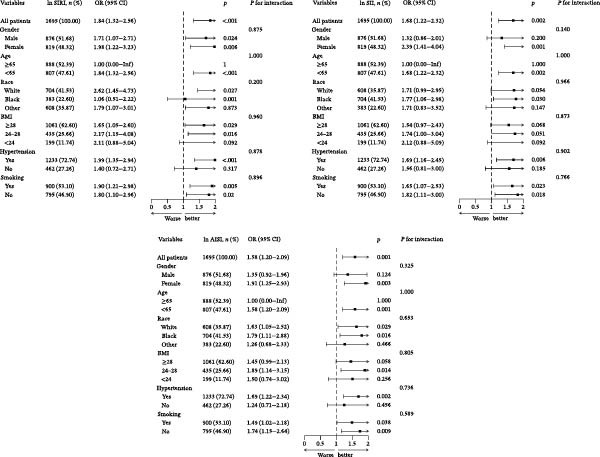
Subgroup analysis for the association between ln SIRI, ln SII, and ln AISI with the prevalence of DFU.

Subgroup analyses stratified by age, gender, race, BMI, smoking status, and hypertension demonstrated consistent associations across all subgroups, indicating the robustness of these relationships without significant effect modification by these demographic and clinical factors (Figure 3).

### 3.2. Clinical Validation Study Findings

The retrospective validation case–control study comprised 102 participants, including 34 DFU cases and 68 diabetic controls without DFU. The DFU group (mean age 64.03 ± 13.31 years) and non‐DFU group (mean age 67.54 ± 10.94 years) showed significant differences in several clinical parameters. DFU patients exhibited elevated levels of CRP, hemoglobin, WBCs, neutrophils, platelets, and albumin, alongside reduced HDL‐cholesterol levels. Importantly, all three systemic inflammatory biomarkers (SIRI, SII, and AISI) were significantly elevated in the DFU group compared to controls (Table 3).

**Table 3:** Clinical characteristics of the retrospective study.

Characteristics	DFU (*n* = 34)	Non‐DFU (*n* = 68)	*p*
Age (years)	64.03 ± 13.31	67.54 ± 10.94	0.158
Gender (%)			0.006
Male	27 (79.4%)	35 (51.5%)	—
Female	7 (20.6%)	33 (48.5%)	—
BMI (kg/m^2^)	24.22 ± 3.43	25.04 ± 3.29	0.244
Duration of diabetes (years)	9.24 ± 1.91	9.03 ± 2.15	0.637
CRP (mg/L)	39.83 ± 64.98	2.71 ± 5.29	<0.001
Hb (g/dL)	114.65 ± 21.80	132.84 ± 15.58	<0.001
WBC (10^9^)	8.78 ± 4.58	6.35 ± 2.37	0.001
Lymphocyte (10^9^)	1.50 ± 0.61	1.72 ± 0.65	0.107
Monocyte (10^9^)	0.55 ± 0.25	0.51 ± 0.16	0.376
Neutrophil (10^9^)	6.56 ± 4.60	3.99 ± 1.95	<0.001
Platelet (10^9^)	256.944 ± 59.97	202.34 ± 61.59	<0.001
Albumin (g/L)	33.90 ± 5.02	39.66 ± 3.61	<0.001
Total cholesterol (mmol/L)	3.91 ± 1.49	3.92 ± 0.28	0.965
HDL‐cholesterol (mmol/L)	0.93 ± 0.27	1.12 ± 14.34	0.001
LDL‐cholesterol (mmol/L)	2.39 ± 0.82	2.38 ± 0.93	0.952
Triglyceride (mmol/L)	1.90 ± 3.26	1.53 ± 0.84	0.389
SIRI	4.49 ± 10.45	1.35 ± 0.96	0.015
SII	1708.80 ±2992.67	527.33 ± 362.87	0.002
AISI	1233.33 ± 2794.05	280.82 ± 228.15	<0.001
ln SIRI	0.70 ± 1.09	0.10 ± 0.64	0.001
ln SII	6.92 ± 0.90	6.08 ± 0.61	<0.001
ln AISI	6.22 ± 1.20	5.36 ± 0.76	<0.001

Abbreviations: AISI, aggregate index of systemic inflammation; SII, systemic immune‐inflammatory index; SIRI, systemic inflammatory response index

Logistic regression analyses in the validation study confirmed strong positive associations between systemic inflammatory biomarkers and DFU prevalence: ln SIRI: OR = 2.51 (95% CI: 1.39–4.54), ln SII: OR = 5.44 (95% CI: 2.48–11.91), and ln AISI: OR = 2.75 (95% CI: 1.59–4.74). In the fully adjusted model (Model 3), which accounted for age, sex, diabetes duration, BMI, and albumin levels, the ORs were as follows: ln SIRI: OR = 2.95 (95% CI: 1.18–7.38), ln SII: OR = 9.98 (95% CI: 2.67–37.26), and ln AISI: OR = 3.12 (95% CI: 1.37–7.10; Table 4).

**Table 4:** Multivariable binary logistic regression of the relationship between ln SIRI, ln SII, and ln AISI and the risk of DFU.

Variables	Model 1		Model 2		Model 3	
	OR (95% CI)	*p*	OR (95% CI)	*p*	OR (95% CI)	*p*
ln SIRI	2.51 (1.39, 4.54)	0.002	3.01 (1.53, 5.93)	0.001	2.95 (1.18, 7.38)	0.021
ln SII	5.44 (2.48, 11.91)	<0.001	11.11 (3.65, 33.88)	<0.001	9.98 (2.67, 37.26)	<0.001
ln AISI	2.75 (1.59, 4.74)	<0.001	3.14 (1.70, 5.82)	<0.001	3.12 (1.37, 7.10)	0.007

*Note:* Model 1, unadjusted model; Model 2, adjusted for sex and age; Model 3, adjusted for sex, age, diabetes duration, BMI, and albumin.

Abbreviations: AISI, aggregate index of systemic inflammation; SII, systemic immune‐inflammatory index; SIRI, systemic inflammatory response index.

**Figure 4:**
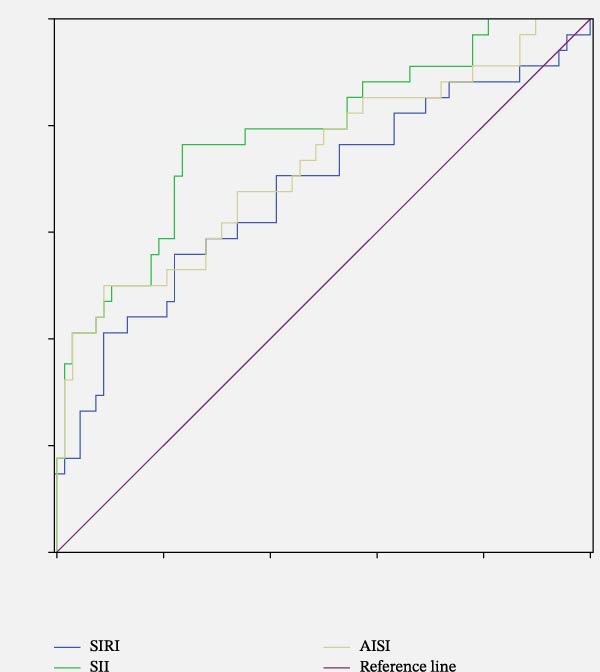
ROC analysis of SIRI, SII, and AISI to indicate DFU.

To preliminarily assess the discriminatory ability of SII, SIRI, and AISI for DFU status, we performed ROC curve analysis (Figure 4). The area under the curve (AUC) values were: SIRI: 0.68 (95% CI: 0.56–0.80, *p* = 0.003), SII: 0.78 (95% CI: 0.69–0.88, *p* < 0.001), and AISI: 0.73 (95% CI: 0.62–0.84, *p* < 0.001), which suggesting modest discriminative capacity in this study (Table 5).

**Table 5:** Comparison of AUC values between three systemic inflammatory biomarkers.

Test	AUC	*p*	95% CI	Threshold	Specificity	Sensitivity
SIRI	0.68	0.003	0.56–0.80	1.78	0.559	0.779
SII	0.78	<0.001	0.69–0.88	695.28	0.765	0.765
AISI	0.73	<0.001	0.62–0.84	583.47	0.500	0.912

Abbreviations: AISI, aggregate index of systemic inflammation; SII, systemic immune‐inflammatory index; SIRI, systemic inflammatory response index.

## 4. Discussion

This study investigated the associations between systemic inflammatory biomarkers, including SIRI, SII, AISI, and DFU through a cross‐sectional analysis of NHANES data and an independent observational retrospective study. Our cross‐sectional analysis revealed significant correlations between SIRI, SII, AISI, and DFU prevalence. RCS analyses demonstrated linear relationships between these inflammatory indices and DFU. Comparative analysis indicated that SIRI exhibited superior predictive capability for DFU risk relative to SII and AISI. The retrospective study further validated the significant associations between all three biomarkers and DFU risk. However, SII showed the strongest association with DFU prevalence among the three indices evaluated.

CBC analysis currently represents one of the most widely utilized methods for inflammatory status assessment. The absolute counts of neutrophils, lymphocytes, monocytes, and platelets, along with derived parametric indices, can serve as valuable systemic inflammatory biomarkers. Neutrophil counts reflect acute inflammatory responses [10]. The chronic hyperglycemic state characteristic of diabetes mellitus impairs neutrophil function, consequently prolonging inflammatory phases and disrupting the delicate balance essential for effective wound healing [11]. Lymphocyte counts play crucial roles in adaptive immunity, with lymphopenia serving as an indicator of immunocompetence [12]. DFU patients exhibit significant oxidative stress in lymphocytes, manifested through reactive oxygen species (ROS) accumulation, membrane damage, increased protein carbonyl groups, and altered superoxide dismutase (SOD) and catalase activities [13]. Platelet counts reflect both coagulation function and inflammatory activity [14]. Enhanced platelet activation not only releases inflammatory mediators but also promotes thrombus formation through adhesion to damaged vascular endothelium, contributing to atherosclerotic progression and local ischemia [15]. Monocyte counts amplify inflammatory cascades via cytokine release and immune cell activation [16]. Impaired wound healing in diabetic patients has been associated with increased monocyte/macrophage infiltration and disrupted transition from pro‐inflammatory to pro‐healing phenotypes [17]. Given the accessibility, reproducibility, and cost‐effectiveness of CBC analysis for inflammatory detection, the identification of novel inflammatory biomarkers derived from CBC parameters has gained considerable research interest in recent years.

Recent research has identified several novel systemic inflammatory biomarkers, including SIRI, SII, and AISI [18]. SIRI, calculated as (neutrophil count × monocyte count)/lymphocyte count, was initially developed in 2016 as a prognostic indicator for survival outcomes in pancreatic cancer patients following chemotherapy [19]. SII, derived from (platelet count × neutrophil count)/lymphocyte count, was first established to predict clinical outcomes in hepatocellular carcinoma patients after curative resection [20]. AISI, computed as (neutrophil count × platelet count × monocyte count)/lymphocyte count, was initially investigated in 2018 for its prognostic value in thoracic surgical patients [21]. These hematological indices, derived from routine CBC parameters, demonstrate enhanced capability in reflecting comprehensive immune‐inflammatory information and provide superior assessment of the balance between inflammatory and immune status [22]. Substantial evidence supports positive associations between SIRI, SII, and AISI, and various chronic inflammatory diseases [23–25].

Accumulating evidence has demonstrated the utility of SIRI, SII, and AISI in evaluating diabetes progression and its associated complications. Wang et al. [26] reported that SII was an independent predictor of the severity of coronary plaque burden in diabetic patients with coronary heart disease. Weighted multiple logistic regression found that with every one‐unit increment in AISI/1000, there was an 88.3% likelihood of type 2 diabetes occurrence (OR: 1.883, 95% CI: 1.38–2.57) [27]. Song et al. [6] demonstrated the levels of SII, SIRI, and AISI were higher in peripheral arterial disease in type 2 diabetic patients, which were independently lined with its clinical severity. Despite these advances, the application of these biomarkers in DFU patients remain limited.

In our cross‐sectional analysis, ln SIRI, ln SII, and ln AISI demonstrated significant positive associations with DFU prevalence: ln SIRI (OR = 2.03, 95% CI: 1.48–2.80), ln SII (OR = 1.78, 95% CI: 1.29–2.44), and ln AISI (OR = 1.70, 95% CI: 1.30–2.23). Compared to the first tertile, the third tertile exhibited substantially increased DFU risk: 107% for ln SIRI, 79% for ln SII, and 103% for ln AISI. Subgroup analyses confirmed the robustness of these associations. Among the three biomarkers, SIRI demonstrated the strongest correlation with DFU risk. Furthermore, the accessibility and cost‐effectiveness of these laboratory‐derived biomarkers enhance their potential clinical utility.

Our retrospective observational study similarly demonstrated significant associations between all three systemic inflammatory biomarkers and the risk of DFU: ln SIRI: OR = 2.51 (95% CI: 1.39–4.54), ln SII: OR = 5.44 (95% CI: 2.48–11.91), and ln AISI: OR = 2.75 (95% CI: 1.59–4.74). Among these, SII showed the strongest association with DFU prevalence. After adjusting for key confounders including age, sex, diabetes duration, BMI, and albumin levels, the associations between these biomarkers and DFU remained consistent in both direction and statistical significance, supporting the robustness of our primary findings. The results of ROC analysis also showed that SII was more reliable than the other two for predicting DFU. Notably, these findings appears inconsistent with our cross‐sectional results, which identified SIRI as the strongest predictor. Methodological differences may explain this discrepancy: the retrospective study utilized clinical data from hospitalized diabetic patients, while the cross‐sectional analysis employed population‐based data from NHANES participants, who generally represent a baseline health state. Hospitalized patients typically experience physiological stress due to infection, trauma, surgical interventions, or chronic disease exacerbations. These divergent population characteristics likely contribute to the observed differences in biomarker performance. This inconsistency should not be interpreted as a methodological limitation but rather as a reflection of biological and clinical complexity. Future investigations should incorporate stratified analyses, standardized measurement protocols, and multivariate modeling approaches to quantify the relative contributions of various factors to these observed differences.

This study employed a combined approach of cross‐sectional analysis and observational retrospective investigation to examine the associations between three systemic inflammatory biomarkers (SIRI, SII, and AISI) and DFU. To the best of our knowledge, this is the first study to evaluate the relationship between these biomarkers and DFU prevalence. Using large‐scale cohort data from the NHANES database, we demonstrated that SIRI, SII, and AISI were significantly associated with DFU. To enhance the robustness of our findings, a clinical retrospective study was additionally conducted. The combination of cross‐sectional and clinical methodologies strengthens the validity and objectivity of the results.

Several limitations of this study warrant acknowledgment. First, due to the cross‐sectional design, causal inferences between systemic inflammatory biomarkers and DFU progression cannot be established. Future longitudinal studies are warranted to examine temporal changes in these biomarkers and their predictive capacity for DFU development. Second, while the cross‐sectional analysis utilized a nationally representative U.S. sample, the retrospective study was based primarily on a Chinese population, necessitating further validation across diverse ethnic and geographic groups. Third, certain variables were collected through questionnaires and self‐reports, which may be subject to recall bias. Fourth, although multiple confounders were adjusted for, residual confounding from unmeasured covariates cannot be fully excluded. Fifth, the number of DFU events in our retrospective study was limited. While our models were adjusted for a minimal set of key confounders, the analysis of multiple correlated inflammatory indices increases the risk of model overfitting and may inflate the precision of the ORs. Our findings, particularly the exact effect sizes, should, therefore, be interpreted with caution and require validation in larger, prospective studies. Moreover, our ROC analyses are conducted in a small, single‐center dataset without internal validation or external replication. Consequently, the proposed cutoff values for SII, SIRI, and AISI are unlikely to be stable or generalizable and serve here primarily as an initial clinically intuitive reference. Our findings regarding specific cutoffs are strictly preliminary and must be validated in larger, prospective studies before any clinical application can be considered.

In conclusion, this study integrated a cross‐sectional analysis of the NHANES database with a clinical retrospective study to evaluate the relationship between systemic inflammatory biomarkers and DFU. The results consistently indicated that elevated levels of SIRI, SII, and AISI were significantly associated with DFU prevalence. Notably, the cross‐sectional analysis identified SIRI as having superior predictive accuracy, whereas the retrospective study suggested that SII may be a more reliable biomarker. These divergent findings highlight the need for further investigation through randomized controlled trials and larger, more diverse studies. Overall, our findings support the potential integration of systemic inflammatory biomarkers into clinical practice for early identification of high‐risk individuals, facilitating timely intervention and improved DFU management.

## Author Contributions

Conceptualization, writing – original draft: Jiezhi Dai. Investigation: Hua Chen and Yu Zhou. Methodology, writing – review and editing: Jiezhi Dai, Yu Zhou, and Hua Chen.

## Funding

The authors have nothing to report.

## Disclosure

All authors have read and approved the manuscript and ensure that this is the case.

## Ethics Statement

The NCHS Ethics Review Board approved the NHANES protocol, and all participants provided informed consent. This study strictly adhered to the ethical standards outlined in the 1964 Declaration of Helsinki and its subsequent revisions. The data were extracted for secondary analysis, obviating the need for additional ethical approval. The clinical retrospective study involving human participants were reviewed and approved by the Ethic Review Board of Shanghai Sixth People’s Hospital. The patients/participants provided their written informed consent to participate in this study. Written informed consent was obtained from the individuals for the publication of any potentially identifiable images or data included in this article.

## Conflicts of Interest

The authors declare no conflicts of interest.

## Supporting Information

Additional supporting information can be found online in the Supporting Information section.

## Supporting information


**Supporting Information** STROBE Statement—Checklist of items that should be included in reports of cross‐sectional studies.

## Data Availability

The data used to support the findings of this study are included within the article, which are available from the corresponding author upon request.
